# On the Diagnostic Value of the Degree of Acidity of the Gastric Juice

**Published:** 1894-03-31

**Authors:** 


### On the Diagnostic Value of the Degree of Acidity of the Gastric Juice.

Very much attention has recently been directed to the acidity of the gastric juice in health and disease. In health the gastric juice is always acid. Prout, in 1824, proved the existence of hydrochloric acid in the gastric juice. Also there is present in the contents of a fresh, healthy stomach lactic acid, which is not a product of glandular secretion, but is a consequence of the normal fermentation of carbo-hydrates, or is brought into the stomach with the food. Ewald and Boas have shown that at the commencement of the digestion of a meal of bread, or meat with bread and potatoes, in a healthy stomach lactic acid or sarco-lactic acid is always present. This is so for about half an hour after the ingestion of food, and this acid disappears as soon as the free hydrochloric acid becomes noticeable. This is begun to be secreted directly food is put into the stomach, but that first formed enters into combination with the albuminous bodies and salts of the food. After their affinities are satisfied free hydrochloric acid can be detected.

The best reagent for the determination of hydrochloric acid is that recommended by Günzburg, composed of phloroglucine two grains, vanilline one grain, and absolute alcohol thirty grains. The reaction is that a splinter dipped in the solution becomes deep red when touched with hydrochloric acid; fluids containing one-twentieth part per 1,000 of free hydrochloric acid will give this reaction.

For lactic acid a good test is to take a mixture of a few drops of carbolic acid and ferric chloride diluted with water to the colour of amethyst-blue, which, in the presence of minute traces of lactic acid, becomes a bright canary yellow. As a rule, the free hydrochloric acid in the gastric contents is between a half and two parts per thousand. After a simple test breakfast the acid is at its maximum in an hour, and in three hours after a dinner. It is stated that with practice the amount of free hydrochloric acid can be easily estimated by noticing the depth of colour given by the Günzburg test. In this case it is best to evaporate a few drops of the test fluid with a few drops of the stomach contents slowly in a white porcelain dish, in which the colour shows up very well.

Great importance has been attached to the presence or absence of free hydrochloric acid in the gastric contents in cases in which the diagnosis of gastric ulcer or gastric carcinoma was doubtful, for it was stated that free hydrochloric acid was never met with in the gastric contents in these two diseases. It has recently, however, been made out that the acid is often absent in cases of heart disease.

Salzer has made a number of experiments, and he finds free hydrochloric acid in about one-third of all the cases he examined. Most of his patients had comparatively good digestive powers. Of the forty-four cases in which it was absent four were examples of carcinoma of the stomach, three were instances of primary gastritis, and a large number suffered from chronic gastritis associated with various conditions, such as valvular disease of the heart, fatty heart, cirrhosis of the liver, and Bright's disease. The large number of cases in which the absence of hydrochloric acid was associated with heart disease show that venous congestion of the stomach impairs the secretion of free hydrochloric acid. Thirty-nine of the patients had moderately good digestion, in spite of the absence of hydrochloric acid. In three cases this absence continued for months.

Thirty-three of his patients had a normal amount of hydrochloric acid in the expressed gastric contents. Of these thirty-three patients seventeen had chronic gastritis, nine had nervous dyspepsia, and five had gastric ulcer.

In eighteen cases absence of hydrochloric acid, alternated with the presence of the normal amount. These patients all suffered either from chronic gastritis or nervous dyspepsia.

In twenty-five of his cases there was an excess of hydrochloric acid in the gastric contents; five of these patients had chronic gastritis, fourteen had nervous dyspepsia, and six had gastric ulcer.

It will be noticed that the main result of these researches is to show that variations in the amount of hydrochloric acid in the gastric juice are wide, and especially they bring out the fact that it is absent oftener than was previously thought.

It is in cases of cancer of the stomach that attention to the acidity has been most frequently directed. Von Jaksch, for instance, tested seventeen cases, and found that in fourteen hydrochloric acid was absent after a test meal, but in three there was plenty of acid present. We may therefore conclude that hydrochloric acid is often absent in cases of cancer of the stomach, and it will be noticed that Salzer found it absent in all the cases he examined. But it is also clear that the absence of the acid is only an aid to diagnosis of cancer of the stomach, for not only may it be sometimes present in instances of this disease, but it is often absent in other conditions.

In chronic ulcer of the stomach Salzer's researches show what Riegel has already pointed out, namely, that the amount of hydrochloric acid is often very greatly increased, usually, according to this author, three or four times. Several authors have recorded cases in which the hyperacidity of the gastric contents has diminished as the disease has progressed.

The condition of the acidity of the contents of the stomach in other gastric disorders still requires much elucidation. We have already mentioned the effect of venous congestion in diminishing the amount of hydrochloric acid secreted. With regard to acute gastritis, very little is known, but it is said that hydrochloric acid is absent. In chronic gastritis, recent observations appear to show that it is possible to distinguish four forms of this disease. According to Ewald they are: (1) Simple gastritis, in which the test breakfast is never followed by increased acidity, the proportion of hydrochloric acid being diminished. (2) An acid gastritis, in which the amount of hydrochloric acid is greatly increased; in other respects the condition is the same as in simple gastritis. (3) Mucous catarrh, in which the acidity is slight and hydrochloric acid is absent. Artificial digestion requires the addition of hydrochloric acid. (4) Atrophic gastritis, in which the contents, after the administration of a test meal, are free from mucous, pepsin, hydrochloric acid, and the milk-curdling ferment.

